# Editorial: Vectors and Vector-Borne Parasitic Diseases: Infection, Immunity, and Evolution

**DOI:** 10.3389/fimmu.2021.729415

**Published:** 2021-07-21

**Authors:** Kokouvi Kassegne, Xiao-Nong Zhou, Jun-Hu Chen

**Affiliations:** ^1^ School of Global Health, Chinese Centre for Tropical Diseases Research, Shanghai Jiao Tong University School of Medicine, Shanghai, China; ^2^ National Institute of Parasitic Diseases, Chinese Center for Diseases Control and Prevention (Chinese Centre for Tropical Diseases Research), National Health Commission of the People's Republic of China (NHC) Key Laboratory of Parasite and Vector Biology, WHO Collaborating Centre for Tropical Diseases, National Center for International Research on Tropical Diseases, Shanghai, China

**Keywords:** vector-borne parasitic diseases, malaria, schistosomiasis, Chagas disease, leishmaniasis, babesiosis, theileriosis

## 

The (re)emergence and burden of vector-borne parasitic diseases (VBPDs) draws attention to vectors with the parasites they carry. These include, but are not limited to: anopheles-borne diseases (e.g., caused by *Plasmodium*); tick-borne diseases (e.g., caused by *Babesia* and *Theileria*); snail-borne diseases (e.g., caused by *Schistosoma*); sand fly-borne diseases (e.g., caused by *Leishmania*); and tsetse fly- or triatomine bug-borne diseases (e.g., caused by *Trypanosoma*)*. *Knowledge of vector and parasite biology is beginning to stimulate new concepts and tools for effective disease control. However, the mechanisms and pathways through which the development of parasites within vectors may enhance vector-mediated immune control or regulate interactions are not properly understood. In addition, the mechanisms by which hosts respond to parasite exposure and acquire immunity remain unclear. A further major challenge in combatting VBPDs is the high variability of a large repertoire of genes in the genome of a single parasite. The 14 articles of this themed Research Topic highlight the latest advances regarding vectors and VBPDs: infection, immunity, and evolution.

Understanding the immunological machinery of host-parasite interactions will advance knowledge of VBPD mechanisms to track relevant immune and parasitic molecules that ensure protection or drive disease. Induction of humoral immunity is critical for clinical protection against infection. Antibody levels targeting antigens are predictive of infection and lay the basis for the identification of biomarkers of exposure/candidate antigens for serodiagnostics or vaccines. In this collection, Hou et al. advance knowledge of B-cell epitopes of 37 erythrocyte invasion-associated antigens of *Plasmodium falciparum*, which have been tested in clinical settings as vaccine candidates. They find that most immunogenic epitopes are predominantly located in the low-complexity regions of the proteins containing repetitive and/or glutamate-rich motifs, and the epitope-derived specific antibodies could not inhibit erythrocyte invasion (Hou et al.). These indicate that immune responses could be driven away from functional domains of *P. falciparum* proteins, which is an instructive finding for the rational design of blood-stage malaria vaccine candidates. In an *in vitro* and *in vivo* study, Wang et al. evaluate the potential applications of BmSP44, an erythrocyte invasion-associated merozoite surface antigen of *Babesia microti*. The authors validate BmSP44 as a dominant immunogen candidate and demonstrate that it is a secreted protein localized in the parasite’s cytoplasm. They discover that recombinant BmSP44 antisera can attenuate parasitaemia in infected mice and, more importantly, is associated with clinical protection against *B. microti* infection in immunized mice. This is reflected in a Th1/Th2 mixed immune response with significantly elevated levels of interferon-gamma (IFN-γ) and interleukin (IL)-10 during the early stage of infection (Wang et al.).

Pro- and anti-inflammatory cytokines are important mediators of cellular immunity and are associated with parasitic disease outcomes. It is a generally accepted observation that clinical symptoms develop as a result of immunopathology involving dysregulation of immune mediator balance in favor of pro-inflammatory mediators. Therefore, understanding the role of immune regulators in the establishment of protective immunity or asymptomatic infections is critical. In this regard, Frimpong et al. determine the association of pro-inflammatory mediators including tumor necrosis factor-alpha (TNF-α), IFN-γ, IL-6, IL-12p70, IL-17A and granzyme B, and the cytokines IL-4 and IL-10 with the development of asymptomatic malaria. The authors find that neither microscopic nor submicroscopic asymptomatic infection is polarized toward a pro- or ‘‘anti’’-inflammatory response, but rather by a balanced inflammatory response (ratio of IFN-γ/IL-10, TNF-a/IL-10, IL-6/IL-10 as well as IFN-γ/IL-4 and IL-6/IL-4 not significantly different). This advances knowledge that asymptomatic malaria infections result in increased plasma levels of both pro- and ‘‘anti’’-inflammatory cytokines relative to uninfected persons, which explains in part the lack of clinical symptoms. However, not much evidence has been forthcoming in favour of the hypothesis that IL-4 and IL-10 may be anti-inflammatory or regulatory cytokines. Unveiling the protective immune response to VBPDs is critical for a rational design of vaccines. Soto et al. add to our understanding of the role of basic leucine zipper transcription factor ATF-like 3 (Batf3) in the generation of type 1 immunity against parasitic infections. The authors demonstrate that Batf3-deficient mice are unable to control hepatic parasitosis as opposed to wild-type C57BL/6 mice. In addition, the impaired microbicide capacities of *Leishmania infantum*-infected macrophages from Batf3-deficient mice correlates with a reduction of parasite-specific IFN-γ production.

*Trypanosoma cruzi* is an intracellular protozoan that can reside within different tissues, evading host immunity and allowing progression towards chronic stages of infection. Such intracellular parasitism triggers strong cellular immunity that, besides being necessary to limit infection, is not sufficient to eradicate parasites from tissues. Two *in vivo* studies published in this collection provide novel insights onto vaccine candidates by *T. cruzi* infection. Antonoglou et al. explore new strategies for a differential immune response for vaccines against Chagas disease using an immunogenic chimeric molecule—NCz-SEGN24A. The authors find that NCz-SEGN24A enhances significant production of specific IgG titers in immunized mice, with significant specific cell-mediated immune responses. More importantly, the immunogen confers protection against infection in immunized mice, with 100% survival maintained in mice challenged with trypomastigotes of *T. cruzi* (Antonoglou et al.). Such a finding encourages the testing of mutated superantigens fused to specific antigens as immune modulators. In a mouse model study, Cerny et al. demonstrate that a *Salmonella*-based therapeutic DNA vaccine that combines Cruzipain (Cz) and Chagasin (Chg) provides improved protection than monocomponent therapeutic vaccines against each. The authors find that the bicomponent vaccine could significantly (i) increase the titers of antigen- and parasite-specific antibodies, and (ii) trigger a robust cellular response with IFN-γ secretion that rapidly reduces the parasitaemia during the acute phase and decreases the tissue damage in the chronic stage of the infection. Such a bicomponent vaccine strategy could be an effective tool to ameliorate the pathology associated to Chagas disease. Meanwhile, the presence of intracellular protozoa among immunocompromised HIV-infected individuals increases the incidence of severe disease. In an *in vitro* study, Urquiza et al. show that the pathogenesis of HIV-*T. cruzi* coinfection in astrocytes: (i) leads to an oxidative misbalance mechanism which increases mitochondrial and cellular reactive oxygen species (ROS) production, and (ii) boosts *T. cruzi* multiplication towards severe meningoencephalitis in the central nervous system (CNS). Findings from their study imply that ROS production drives astrocyte infection by *T. cruzi*, and involves ROS production from HIV-exposed astrocytes during coinfection, contributing to parasite persistence and CNS pathology (Urquiza et al.). These insights shed light on the pathogenesis of neurologic Chagas disease and will inform the design of future parasite control strategies.

In another *in vivo* study conducted with *L. major*, Keshavarzian et al. evaluate *Iranian Lizard Leishmania* (ILL) mixed with CpG-ODN as a bicomponent candidate vaccine against experimental murine leishmaniasis. The authors find that ILL+CpG ensures protection against the development of dermal lesions in immunized mice with a significant reduction in the parasite load in comparison to control groups. In addition, this is associated with higher production of IFN-γ, as well as a reduction in IL-4 levels and arginase activity. These findings indicate that ILL, with an appropriate adjuvant, might be suitable for use as a vaccine against leishmaniasis. Likewise, Zhang et al. utilize a recently developed humanized mouse that has been used to assess immune responses against an attenuated C9 parasite clone (C9-M) - carrying a single insertion disrupting the open reading frame of PF3D7_1305500 - of *P. falciparum*. Zhang et al. identify that the attenuated falciparum parasite induces distinct patterns of cytokine production in these humanized mice to evade residual protective non-adaptive immune responses. Their study unveils a valuable way to explore the role of C9 mutation in the growth and survival of parasite mutants and their response to the host’s immune responses. This mouse might, therefore, help identify novel targets for antimalarial chemotherapy.

An *in vitro* and *in vivo* study by Huang et al. in this collection provides the first direct evidence that a natural flavonol compound—myricetin, possesses potent anti-schistosome activities. The authors find that myricetin could exhibit dose and time-dependent anthelmintic effects on *Schistosoma japonicum* and inhibited female spawning, suggesting that it could be further explored as a therapeutic agent. Their study also offers new insights into the mechanisms of action of myricetin by revealing that it attenuates hepatic fibrosis via modulating transforming growth factor beta (TGFβ) 1 and Akt signaling, and shifting the Th1/Th2 balance in *S. japonicum*-infected mice (Huang et al.).

The high variability in the clinical course of VBPDs in patients raises fundamental questions about parasitic factors that are critical in regulating pathogenesis, disease severity, or drug resistance. In this collection, two exciting studies apply genomic approaches to dissect the genetic backgrounds of isolate parasite populations for malaria and theileriosis surveillance.

Firstly, Kassegne et al. provide the first genotyping of single nucleotide polymorphisms and local-specific signals of selection in ten clinical *P. falciparum* isolates from Togo. The authors find relatively high genome-wide diversity and recent population expansion of *P. falciparum* in Togo. Against this background, they identify a total of 383 genes under local-specific signals of selection. Importantly, host immunity is found to be the major selective agent on antigen genes, including membrane and surface proteins implicated in merozoite invasion and malaria severity, respectively. Interrestingly, they find that Togo *P. falciparum* is under no serious antimalarial drug selection (Kassegne et al.). Secondly, Roy et al. address major questions regarding the population structure and genetic diversity of *Theileria annulate* in field isolates and the impact of the theileriosis vaccine currently being used in India. Their findings highlight: (i) high transmission intensity and abundance of ticks in India in compliance with high genetic variation between the tick-borne parasite populations; and (ii) limited genetic diversity in the vaccine isolates in the country, which suggests efficacy of the schizont stage theileriosis vaccine. The authors also identify a new panel of markers that could be helpful for surveillance of this tick-borne parasite (Roy et al.). However, the low diversity in the isolates from vaccinated individuals advocates improving the current vaccine, possibly by increasing its heterozygosity.

Understanding how the mechanisms and pathways through which the development of parasites within vectors may enhance vector-mediated immune control or regulate interactions will provide critical insights into relevant molecules that secure protection in vectors.

Sharma et al. investigate how *P. vivax* alters gut-microbiota and antiplasmodial immunity and impacts tripartite *Plasmodium*-mosquito-microbiota interactions in the gut lumen. In a metagenomics and RNAseq study, Sharma et al. provide evidence that, during the preinvasive phase, *P. vivax* suppresses midgut-microbiota, which likely negates the impact of mosquito immunity and, in turn, may enhance the survival of *P. vivax*. The authors conclude that the detection of sequences matching mosquito-associated *Wolbachia* would open a new inquiry for its exploration as an agent for “paratransgenesis-based” mosquito control. Likewise, Hu et al. advance our understanding of the critical events during schistosome infection by demonstrating changes in the gut microbiome composition during schistosomiasis progression, the functional interactions between the gut bacteria and *S. japonicum* infection in mice, and the dynamic metabolite changes in the host. The authors observe a decrease in richness and diversity, as well as differed composition, of the gut microbiota in infected status versus uninfected status. They also discover metabolic biomarkers including phosphatidylcholine and colfosceril palmitate in serum, as well as xanthurenic acid, naphthalenesulfonic acid, and pimelylcarnitine in the urine (Hu et al.). These biomarkers could serve as new targets for early diagnosis and prognostic purposes following *S. japonicum* infection.

Collectively, this themed Research Topic advances our knowledge of the molecular and cellular processes involved in parasitic disease susceptibility, which may pave the way for vector control interventions as well as diagnosis, therapy, and rational design of vaccines for VBPDs.

## Author Contributions

KK and J-HC drafted the manuscript. All authors contributed to the article and approved the submitted version.

## Funding

This work was supported by the National Research and Development Plan of China (Grant No. 2018YFE0121600), the National Sharing Service Platform for Parasite Resources (Grant No. TDRC-2019-194-30), and the National Natural Science Foundation of China (Grant No. 81101266). The funding bodies had no role in the design of the study, in collection, analysis, and interpretation of data, or in writing the manuscript.

## Conflict of Interest

The authors declare that the research was conducted in the absence of any commercial or financial relationships that could be construed as a potential conflict of interest.

